# Application of Improved 5th-Cubature Kalman Filter in Initial Strapdown Inertial Navigation System Alignment for Large Misalignment Angles

**DOI:** 10.3390/s18020659

**Published:** 2018-02-23

**Authors:** Wei Wang, Xiyuan Chen

**Affiliations:** Key Laboratory of Micro-Inertial Instrument and Advanced Navigation Technology, Ministry of Education, School of Instrument Science and Engineering, Southeast University, Nanjing 210096, China; 230159566@seu.edu.cn

**Keywords:** initial alignment, large misalignment angle, fifth-degree cubature Kalman filter, fading memory index weighting

## Abstract

In view of the fact the accuracy of the third-degree Cubature Kalman Filter (CKF) used for initial alignment under large misalignment angle conditions is insufficient, an improved fifth-degree CKF algorithm is proposed in this paper. In order to make full use of the innovation on filtering, the innovation covariance matrix is calculated recursively by an innovative sequence with an exponent fading factor. Then a new adaptive error covariance matrix scaling algorithm is proposed. The Singular Value Decomposition (SVD) method is used for improving the numerical stability of the fifth-degree CKF in this paper. In order to avoid the overshoot caused by excessive scaling of error covariance matrix during the convergence stage, the scaling scheme is terminated when the gradient of azimuth reaches the maximum. The experimental results show that the improved algorithm has better alignment accuracy with large misalignment angles than the traditional algorithm.

## 1. Introduction

Initial alignment is the basis for obtaining accurate misalignment angles of Strapdown Inertial Navigation Systems (SINS), and its accuracy and efficiency will directly affect the SINS performance [1,2]. In an application environment with large misalignment angles and moving bases, the linear initial alignment algorithm is greatly restricted [3], while nonlinear algorithms have been increasingly used for initial alignment [4,5].

In order to apply the linear Kalman filter to a nonlinear system, the Extended Kalman Filter (EKF) [6] truncates the Taylor series expansion of the nonlinear function. The accuracy of EKF is low for strongly nonlinear systems, and there may be a problem in that the Jacobi matrix cannot be solved [7]. An Unscented Transformation (UT) is used in the Unscented Kalman Filter (UKF) [8] to replace the local linearization for obtaining the first two moments of the states. Although the precision of UKF is higher than EKF, there is a “dimension disaster” problem [9] in high dimensional applications of UKF, and parameter adjustment is needed when using UKF. Spherical Radial Cubature Rule (SRCR) is used in the third-degree CKF (hereinafter referred to as CKF3) to approximate the nonlinear integral and obtain the two moments of the states, which can achieve third-order accuracy [10]. CKF has a better numerical stability than UKF, and has been widely applied [11,12]. In order to further improve the accuracy of the CKF3, fifth-degree CKFs (hereinafter referred to as CKF5) [13,14] have been applied gradually [15]. CKF5 has problems of numerical stability and big calculation [16], and in this sense, CKF5 is more suitable for low dimensional systems. The adaptive Kalman filter is an effective method to restrain the divergence and improve the accuracy of filtering. The main idea of the adaptive Kalman filter is to scale the part of error covariance matrix or system noise matrix by calculating the ratio of matrix ranks of actual updating and filter updating [17,18]. The traditional method for estimating the updating covariance is the open window method, which uses the mean value of historical data and not takes into account the different weights of new and old updates. The accuracy and real-time performance of the traditional adaptive Kalman filter should be improved. Combining EKF and adaptive filtering [19], the update covariance matrix is directly introduced into the calculation of gain matrix, which improves the utilization rate of updates. However, EKF cannot effectively meet the accuracy requirement of the nonlinear initial alignment with large misalignment angles. Adaptive filtering is introduced into Unscented Particle Filter (UPF) [20], and the efficiency of convergence can be improved by partially scaling the error covariance matrix. Nevertheless, the precision of in-motion and nonlinear initial alignment with large misalignment angles should be further verified experimentally. The 5-th CKF is directly applied to the initial alignment [12], but experiments on initial alignment with large misalignment angles are lacking. The singular value decomposition can improve the robustness of the initial alignment algorithm [21], but there is no verification of actual data except for simulations.

In order to improve the efficiency and accuracy of the 5-th CKF in initial alignment situations, an entire scaling of the error covariance matrix method with the ratio of matrix ranks of actual updates and filtered updates is introduced in this paper. To solve the problem of oscillation or divergence of improved 5-th CKF caused by excessive scaling of the error covariance matrix, the scaling is terminated when the gradient of the azimuth error reaches the maximum. The improved algorithm needs to store the historical IMU data, GNSS data and the calculated azimuth misalignment angle data, and then recalculate it for alignment. To improve the stability of the 5-th CKF algorithm, the Singular Value Decomposition (SVD) algorithm is used instead of the Cholesky’s method in the matrix decomposition. The large misalignment angle model is simplified in this paper, which means that only the velocity errors and the misalignment angles are used as the states. The reduction of the states has the advantages of requiring less calculation and better calculation stability, which can reduce the chances of filter divergence.

In this paper, the nonlinear model of initial alignment with large misalignment angles is first analyzed and a simplified alignment model with 6-dimensional state variables is proposed. Secondly, the traditional CKF5 algorithm is briefly described in this paper, and then the improved algorithms based on fading factor for the updating covariance matrix, short-term scaling of the error covariance matrix and SVD decomposition are introduced. Finally, the effectiveness of the improved algorithms is verified by simulation experiments and the dynamic vehicle experiments.

## 2. Nonlinear Model of Initial Alignment with Large Misalignment Angle

The SINS updating algorithm includes attitude-updating, speed-updating and position-updating, and the corresponding differential equations are expressed with Equations (1) to (3) [22]:

(1) $${\dot{C}}_{b}^{n}=C_{b}^{n}(\omega_{nb}^{b}\times ),$$

(2) $${\dot{v}}^{n}=C_{b}^{n}f_{sf}^{b}-(2\omega_{ie}^{n}+\omega_{en}^{n})\times v^{n}+g^{n},$$

(3) $$\left[\begin{array}{l} \dot{L}\\ \dot{\lambda }\\ \dot{h} \end{array}\right]=\left[\begin{array}{ccc} 0 & 1/R_{Mh} & 0\\ \sec L/R_{Nh} & 0 & 0\\ 0 & 0 & 1 \end{array}\right]v^{n}.$$

where $C_{b}^{n}$ is the direction cosine matrix of the attitude converting from the measurement reference frame (abbreviated as *b* frame) to the navigation reference frame (abbreviated as *n* frame), $\omega_{nb}^{b}=\omega_{ib}^{b}-\left(\omega_{ie}^{b}+\omega_{en}^{b}\right)$ is the rotational angular rate of *b* frame relative to *n* frame, *i* is the inertial reference frame, ($\omega_{nb}^{b}\times )$ is the antisymmetric matrix of $\omega_{nb}^{b}$, the vector $\nu^{n}={\left[\nu_{E}\;\nu_{N}\;\nu_{U}\right]}^{T}$ expresses the eastern velocity, northern velocity and vertical velocity, respectively, $f_{sb}^{b}$ is the output of the accelerometer, *L* is the latitude of the location of carrier, *λ* is the longitude of the location of carrier, *h* is the height of the location of carrier, *R_Mh_* is the radius of meridian of the location of carrier and *R_Nh_* is the radius of prime vertical circle of the location of carrier. Assuming *n*′ and *n* are the calculation reference frame and ideal navigation reference frame, respectively, the misalignment (expressed as Euler angle) between the two navigation reference frames can be expressed as:

(4) $$\dot{\phi }=C_{\omega }^{-1}\omega_{nn^{\prime }}^{n^{\prime }},$$

(5) $$\omega_{nn^{\prime }}^{n^{\prime }}=(I-C_{n}^{n^{\prime }}){\hat{\omega }}_{in}^{n}+C_{n}^{n^{\prime }}\delta \omega_{in}^{n}-C_{b}^{n^{\prime }}(\varepsilon^{b}+w_{g}^{b}),$$

where $\omega_{{nn}^{\prime }}^{n^{\prime }}$ is the relative rotational angular rate between the *n* reference frame and *n*′ reference frame, *ε^b^* is the random constant drift of Fiber Optic Gyroscope (FOG) and $w_{g}^{b}$ is the random drift of FOG. $C_{\omega }^{-1}$ and $C_{n}^{n^{\prime }}$ can be expressed as:

(6) $$C_{\omega }^{-1}=\left[\begin{array}{ccc} \cos\phi_{y} & 0 & \sin\phi_{y}\\ \sin\phi_{x}\sin\phi_{y}/\cos\phi_{x} & 1 & -\sin\phi_{x}\cos\phi_{y}/\cos\phi_{x}\\ -\sin\phi_{y}/\cos\phi_{x} & 0 & \cos\phi_{y}/\cos\phi_{x} \end{array}\right]$$

(7) $$C_{n}^{n^{\prime }}\approx \left[\begin{array}{ccc} \cos\phi_{y}\cos\phi_{z}-\sin\phi_{x}\sin\phi_{y}\sin\phi_{z} & \cos\phi_{y}\sin\phi_{z}+\sin\phi_{x}\sin\phi_{y}\sin\phi_{z} & -\cos\phi_{x}\sin\phi_{y}\\ -\cos\phi_{x}\sin\phi_{z} & \cos\phi_{x}\cos\phi_{z} & \sin\phi_{x}\\ \sin\phi_{y}\cos\phi_{z}+\sin\phi_{x}\cos\phi_{y}\sin\phi_{z} & \sin\phi_{y}\sin\phi_{z}-\sin\phi_{x}\cos\phi_{y}\cos\phi_{z} & \cos\phi_{x}\cos\phi_{y} \end{array}\right].$$

Under the reference frame, Equation (2) can be written as:

(8) $${\dot{\hat{v}}}^{n^{\prime }}=C_{b}^{n^{\prime }}{\hat{f}}_{sf}^{b}-(2{\hat{\omega }}_{ie}^{n}+{\hat{\omega }}_{en}^{n})\times {\hat{v}}^{n}+{\hat{g}}^{n}.$$

Subtracting Equation (2) from Equation (8) and neglecting the calculation error of gravitational acceleration and higher-order terms, we obtain:

(9) $$\delta {\dot{v}}^{n}=[I-{\left(C_{n}^{n^{\prime }}\right)}^{T}]C_{b}^{n^{\prime }}{\hat{f}}_{sf}^{b}+{\left(C_{n}^{n^{\prime }}\right)}^{T}C_{b}^{n^{\prime }}\nabla^{b}-(2{\hat{\omega }}_{ie}^{n}+{\hat{\omega }}_{en}^{n})\times \delta v^{n},$$

where ${\hat{\nu }}^{n^{\prime }}=\nu^{n}+\delta \nu^{n},\;{\hat{f}}_{sf}^{b}=f_{sf}^{b}+\delta f_{sf}^{b},\;\nabla^{b}=\delta f_{sf}^{b}$, and we have:

(10) $${\dot{\varepsilon }}^{b}=0,$$

(11) $${\dot{\nabla }}^{b}=0.$$

The purpose of initial alignment is to quickly identify the misalignment angles. It is difficult to estimate the errors of inertial sensors accurately, and that is not the purpose of initial alignment, then the errors of *ε^b^* and ∇*_b_* can be regarded as system noises. Therefore, the states used in initial alignment are misalignment angles *φ* and the velocity error *δν^n^*, which can reduce the dimensions of the filter, and thus reduce the computation and improve the numerical stability of CKF5. In order to simplify the filtering error equation, a SINS nonlinear error model which can reflect the characteristics of SINS errors has important practical value in the initial alignment [20]. When the SINS and GNSS are integrated for initial alignment under moving base conditions, GNSS can provide relatively accurate velocity and location information, then Equations (4) and (9) can be simplified as:

(12) $$\dot{\phi }=C_{\omega }^{-1}[(I-C_{n}^{n^{\prime }}){\hat{\omega }}_{in,g}^{n}-\varepsilon^{n^{\prime }}]+C_{\omega }^{-1}C_{b}^{n^{\prime }}w_{g}^{b},$$

(13) $$\delta {\dot{v}}^{n}=[I-{\left(C_{n}^{n^{\prime }}\right)}^{T}]C_{b}^{n^{\prime }}{\hat{f}}_{sf}^{b}+{\left(C_{n}^{n^{\prime }}\right)}^{T}\nabla^{n^{\prime }}+{\left(C_{n}^{n^{\prime }}\right)}^{T}C_{b}^{n^{\prime }}w_{a}^{b},$$

where $w_{a}^{b}$ is the random drift of accelerometer, ${\hat{\omega }}_{in,g}^{n}$ is the angular velocity of the Earth determined by the information from GNSS. With the difference between the velocity of SINS measured by GNSS and the calculated velocity working as the measurement of *z*, the nonlinear filtering equations of initial alignment with large misalignment angles can be expressed as:

(14) $$\left\{ \begin{array}{l} \dot{x}=f(x)+g(x)w,\\ z=Hx+v, \end{array}\right.$$

where *x* = [***φ*** (***δν****^n^*)*^T^*], $w=\left[{\left(w_{g}^{b}\right)}^{T}{\left(w_{a}^{b}\right)}^{T}\right]$, H = [**0**_3×3_ ***I***_3×3_], ***ν*** is the error vector of measurement and the function of *f* can be obtained from Equation (12). The model of initial alignment (Equations (12) and (13)) takes into account of the moving base situation, so the estimation of the initial alignment angles will theoretically not be affected by the line movement and angle movement of SINS.

## 3. CKF5 and Improved CKF5

### 3.1. CKF5 Algorithm

For the integral of the global nonlinear function ***I***(*f*) and variable *x* = *ry* in the spherical-radial coordinate system, ***I***(*f*) can be written as:

(15) $$I(f)=\int_{0}^{\infty }\int_{U_{n}}f(ry)r^{n-1}e^{-r^{2}}d\sigma (y)dr,$$

where *δ^T^δ* = 1, *r* ∈ [0, ∞), *U_n_* is n-dimensional unit hypersphere and *σ*(*g*) is the element of *U_n_*. ***I***(*f*) can be divided into two parts, spherical integral *S*(*r*) and radial integral *R*:

(16) $$\left\{ \begin{array}{l} S(r)=\int_{U_{n}}f(ry)d\sigma (y),\\ R=\int_{0}^{\infty }S(r)r^{n-1}e^{-r^{2}}dr. \end{array}\right.$$

The numerical integration is approximated with the summation of weighted sigma points in the algorithm of CKF, then Equation (16) can be expressed as:

(17) $$\left\{ \begin{array}{l} S(r)\approx \sum_{j=1}^{N_{s}}\omega_{s,j}f(ry_{j}),\\ R\approx \sum_{i=1}^{N_{r}}\omega_{r,i}f(r_{i}). \end{array}\right.$$

Then:

(18) $$I(f)\approx \sum_{i=1}^{N_{r}}\sum_{j=1}^{N_{s}}\omega_{r,i}\omega_{s,j}f(r_{i}y_{j}).$$

The m-order approximation of *S*(*r*) can be written as:

(19) $$S=\left\{ \begin{array}{l} \frac{2\prod_{i=1}^{n}\Gamma [(d_{i}+1)/2]}{\Gamma [(\left|d\right|+n)/2]},\;\left\{d_{i}\right\}\;\mathrm{are}\;\mathrm{even}\;\mathrm{numbers},\\ 0,\;\mathrm{at}\;\mathrm{least}\;\mathrm{one}\;\mathrm{odd}\;\mathrm{number}\;\mathrm{in}\;\left\{d_{i}\right\}, \end{array}\right.$$

where *d* = [*p*_1_,*p*_2_,…,*p_n_*], $\left|d=\overset{n}{\underset{i=1}{\sum }}d_{i}\right|$, |d|≤m, and Γ(g) is the gamma function. Note that when *m* = 5, the value of *S* is calculated with $f_{1}(y)=1,\;f_{2}(y_{1})=y_{1}^{2},\;f_{3}(y_{1})=y_{1}^{4}$ and $f_{4}(y_{1},y_{2})=y_{1}^{2}y_{2}^{2}$. The points set on the *U_n_* is defined as:

(20) $$\left\{ \begin{array}{l} {\left[e\right]}_{n}=\left\{\pm {\left\{e_{i}\right\}}_{i=1}^{n}\right\},\\ {\left[s\right]}_{n}=\left\{\pm {\left\{s_{i}\right\}}_{i=1}^{n(n-1)}\right\}, \end{array}\right.$$

where ${\left\{e_{i}\right\}}_{i=1}^{n}$ and ${\left\{s_{i}\right\}}_{i=1}^{n(n-1)}$ are defined as:

(21) $$\left\{ \begin{array}{l} {\left\{e_{i}\right\}}_{i=1}^{n}=\left\{e_{1},e_{2},\ldots ,e_{n}\right\},\\ {\left\{s_{i}\right\}}_{i=1}^{n(n-1)}=\left\{(e_{k}\pm e_{l})/\sqrt{2}:\;k,l=1,2,\ldots ,n.\;\mathrm{and}\;k<l\right\}, \end{array}\right.$$

where $e_{i}$ is the *i*th column of identity matrix $I_{n\times n}$. Then the Equation (19) can be expressed as:

(22) $$S(r)\approx \omega_{1}\sum_{i=1}^{2n}f(r{\left[e\right]}_{i})+\omega_{2}\sum_{i=1}^{2n(n+1)}f(r{\left[s\right]}_{i}),$$

where $\omega_{1}=\frac{(4-n)A_{n}}{2n(n+2)}$, $\omega_{2}=\frac{A_{n}}{n(n+2)}$ and $A_{n}=\left\{{\left[a\right]}_{n},{\left[s\right]}_{n}\right\}$. For the symmetry, $S_{1}(r)=1,\;S_{2}(r)=r^{2}$ and $S_{3}(r)=r^{4}$ are needed to calculated in the solution of 5-order integral of S(r). Then

(23) $$\left\{ \begin{array}{l} \omega_{r,1}r_{1}^{0}+\omega_{r,2}r_{2}^{0}=\frac{1}{2}\Gamma (n/2),\\ \omega_{r,1}r_{1}^{2}+\omega_{r,2}r_{2}^{2}=\frac{1}{2}\Gamma (n/2+1)=\frac{n}{4}\Gamma (n/2),\\ \omega_{r,1}r_{1}^{4}+\omega_{r,2}r_{2}^{4}=\frac{1}{2}\Gamma (n/2+2)=\frac{1}{2}(\frac{1}{2}n+1)(n/2)\Gamma (n/2). \end{array}\right.$$

Let $r_{1}=0$, then we have:

(24) $$\left\{ \begin{array}{l} r_{2}=\sqrt{\frac{1}{2}n+1},\\ \omega_{r,1}=\frac{1}{\left(n+2\right)}\Gamma (n/2),\\ \omega_{r,2}=\frac{n}{2(n+2)}\Gamma (n/2). \end{array}\right.$$

Then it follows from Equations (19) and (23) that the integrated result of Equation (18) can be obtained. The algorithm flow of CKF5 is shown as follows:

Prediction phase:

1. Let $x_{k-1}\sim N(x_{k-1};\;{\hat{x}}_{k-1|k-1},\;P_{k-1|k-1})$ be the estimated state at time k−1 and decompose $P_{k-1|k-1}$ with Cholesky’s method:
  (25) $$P_{k-1|k-1}=S_{k-1|k-1}{S_{k-1|k-1}}^{T}.$$
2. Compute cubature points:
  (26) $$\left\{ \begin{array}{l} x_{1}=S_{k-1|k-1}\cdot 0+{\hat{x}}_{k-1|k-1},\\ x_{2}=S_{k-1|k-1}\sqrt{n+2}\cdot s_{j}^{+}+{\hat{x}}_{k-1|k-1},\\ x_{3}=-S_{k-1|k-1}\sqrt{n+2}\cdot s_{j}^{+}+{\hat{x}}_{k-1|k-1},\\ x_{4}=S_{k-1|k-1}\sqrt{n+2}\cdot s_{j}^{-}+{\hat{x}}_{k-1|k-1},\\ x_{5}=-S_{k-1|k-1}\sqrt{n+2}\cdot s_{j}^{-}+{\hat{x}}_{k-1|k-1},\\ x_{6}=S_{k-1|k-1}\sqrt{n+2}\cdot e_{j}+{\hat{x}}_{k-1|k-1},\\ x_{7}=-S_{k-1|k-1}\sqrt{n+2}\cdot e_{j}+{\hat{x}}_{k-1|k-1}, \end{array}\right.$$
  where $\left\{s_{j}^{+}\right\}≜\left\{\sqrt{\frac{1}{2}}(e_{k}+e_{l}):\;k<l,\;k,l=1,2,\ldots ,n\right\}$ and $\left\{s_{j}^{-}\right\}≜\left\{\sqrt{\frac{1}{2}}(e_{k}-e_{l}):\;k<l,\;k,l=1,2,\ldots ,n\right\}$.
3. Compute the predicted mean ${\hat{x}}_{k|k-1}$ and predicted covariance $P_{k|k-1}$:
  (27) $$\begin{array}{l} {\hat{x}}_{k|k-1}=w_{1}f(x_{1})+w_{2}\sum_{j=1}^{n(n-1)/2}\left(f(x_{2})+f(x_{3})\right)\\ +w_{2}\sum_{j=1}^{n(n-1)/2}\left(f(x_{4})+f(x_{5})\right)+w_{3}\sum_{j=1}^{n}\left(f(x_{6})+f(x_{7})\right), \end{array}$$
  (28) $$\begin{array}{l} P_{k|k-1}=w_{1}f(x_{1})f{\left(x_{1}\right)}^{T}+w_{2}\sum_{j=1}^{n(n-1)/2}\left(f(x_{2})f{\left(x_{2}\right)}^{T}+f(x_{3})f{\left(x_{3}\right)}^{T}\right)\\ +w_{2}\sum_{j=1}^{n(n-1)/2}\left(f(x_{4})f{\left(x_{4}\right)}^{T}+f(x_{5})f{\left(x_{5}\right)}^{T}\right)+w_{3}\sum_{j=1}^{n}\left(f(x_{6})f{\left(x_{6}\right)}^{T}+f(x_{7})f{\left(x_{7}\right)}^{T}\right)\\ -{\hat{x}}_{k|k-1}\cdot {{\hat{x}}_{k|k-1}}^{T}+Q_{k-1}, \end{array}$$
  where $w_{1}=\frac{2}{n+2}$, $w_{2}=\frac{1}{{\left(n+2\right)}^{2}}$, $w_{3}=\frac{4-n}{2{\left(n+2\right)}^{2}}$ and $Q_{k-1}$ is system noise covariance matrix.

Update phase:

4. Decompose $P_{k|k-1}$ with Cholesky’s method:
  (29) $$P_{k|k-1}=S_{k|k-1}{S_{k|k-1}}^{T}.$$
5. Compute cubature points:
  (30) $$\left\{ \begin{array}{l} x_{1}^{\prime }=S_{k|k-1}\cdot 0+{\hat{x}}_{k|k-1},\\ x_{2}^{\prime }=S_{k|k-1}\sqrt{n+2}\cdot s_{j}^{+}+{\hat{x}}_{k|k-1},\\ x_{3}^{\prime }=-S_{k|k-1}\sqrt{n+2}\cdot s_{j}^{+}+{\hat{x}}_{k|k-1},\\ x_{4}^{\prime }=S_{k|k-1}\sqrt{n+2}\cdot s_{j}^{-}+{\hat{x}}_{k|k-1},\\ x_{5}^{\prime }=-S_{k|k-1}\sqrt{n+2}\cdot s_{j}^{-}+{\hat{x}}_{k|k-1},\\ x_{6}^{\prime }=S_{k|k-1}\sqrt{n+2}\cdot e_{j}+{\hat{x}}_{k|k-1},\\ x_{7}^{\prime }=-S_{k|k-1}\sqrt{n+2}\cdot e_{j}+{\hat{x}}_{k|k-1}. \end{array}\right.$$
6. Compute the estimated measurement ${\hat{z}}_{k|k-1}$, the gain matrix $K_{k}$ and the covariance matrixes of $P_{k|k-1}^{zz}$, $P_{k|k-1}^{xz}$:
  (31) $$\begin{array}{l} {\hat{z}}_{k|k-1}=w_{1}h(x_{1}^{\prime })+w_{2}\sum_{j=1}^{n(n-1)/2}\left(h(x_{2}^{\prime })+h(x_{3}^{\prime })\right)\\ +w_{2}\sum_{j=1}^{n(n-1)/2}\left(h(x_{4}^{\prime })+h(x_{5}^{\prime })\right)+w_{3}\sum_{j=1}^{n}\left(h(x_{6}^{\prime })+h(x_{7}^{\prime })\right), \end{array}$$
  (32) $$\begin{array}{l} P_{k|k-1}^{zz}=w_{1}h(x_{1}^{\prime })h{\left(x_{1}^{\prime }\right)}^{T}+w_{2}\sum_{j=1}^{n(n-1)/2}\left(h(x_{2}^{\prime })h{\left(x_{2}^{\prime }\right)}^{T}+h(x_{3}^{\prime })h{\left(x_{3}^{\prime }\right)}^{T}\right)\\ +w_{2}\sum_{j=1}^{n(n-1)/2}\left(h(x_{4}^{\prime })h{\left(x_{4}^{\prime }\right)}^{T}+h(x_{5}^{\prime })h{\left(x_{5}^{\prime }\right)}^{T}\right)+w_{3}\sum_{j=1}^{n}\left(h(x_{6}^{\prime })h{\left(x_{6}^{\prime }\right)}^{T}+h(x_{7}^{\prime })h{\left(x_{7}^{\prime }\right)}^{T}\right)\\ -{\hat{z}}_{k|k-1}\cdot {{\hat{z}}_{k|k-1}}^{T}+R_{k}, \end{array}$$
  (33) $$\begin{array}{l} P_{k|k-1}^{xz}=w_{1}x_{1}^{\prime }h{\left(x_{1}^{\prime }\right)}^{T}+w_{2}\sum_{j=1}^{n(n-1)/2}\left(x_{2}^{\prime }h{\left(x_{2}^{\prime }\right)}^{T}+x_{3}^{\prime }h{\left(x_{3}^{\prime }\right)}^{T}\right)\\ +w_{2}\sum_{j=1}^{n(n-1)/2}\left(x_{4}^{\prime }h{\left(x_{4}^{\prime }\right)}^{T}+x_{5}^{\prime }h{\left(x_{5}^{\prime }\right)}^{T}\right)+w_{3}\sum_{j=1}^{n}\left(x_{6}^{\prime }h{\left(x_{6}^{\prime }\right)}^{T}+x_{7}^{\prime }h{\left(x_{7}^{\prime }\right)}^{T}\right)\\ -{\hat{x}}_{k|k-1}\cdot {{\hat{z}}_{k|k-1}}^{T}, \end{array}$$
  (34) $$K_{k}=P_{k|k-1}^{xz}{P_{k|k-1}^{zz}}^{-1}.$$
7. Update ${\hat{x}}_{k|k}$ and $P_{k|k}$:
  (35) $$P_{k|k}=P_{k|k-1}-K_{k}P_{k|k-1}^{zz}{K_{k}}^{T},$$
  (36) $${\hat{x}}_{k|k}={\hat{x}}_{k|k-1}+K_{k}(z_{k}-{\hat{z}}_{k|k-1}),$$
  where $z_{k}$ is the measurement at time k.

### 3.2. Improved CKF5 Algorithm

The filtering precision of CKF5 can reach the fourth order, and the accuracy of CKF3 can reach the second order. The amount of sigma points of CKF5 is $2n^{2}+1$, and that of CKF3 is 2n, so the cost of computing of CKF3 is less than that of CKF5. In order to estimate the numerical stability of nonlinear filter, the stability factor can be defined as [23]:

(37) $$I=\sum_{i=1}^{N}\left|\omega_{i}\right|,$$

where $\omega_{i}$ is the weight of sigma point and N is the amount of sigma points. If I=1, the numerical integration has absolute stability theoretically, on the contrary, non-absolute stability for I≠1. When I≠1, the cumulative truncation error may leads to a non-positive error covariance matrix, even further to the error of calculation or divergence. For CKF3, I=1 and for CKF5, I≠1, which means that CKF3 has better numerical stability than CKF5. For CKF5, we have:

(38) $$\begin{array}{ll} I & =\left|\frac{2}{n+2}\right|+\left|\frac{n(n-1)}{{\left(n+2\right)}^{2}}\right|+\left|\frac{n(4-n)}{2{\left(n+2\right)}^{2}}\right|\\ & =\frac{2}{n+2}+\frac{n(n-1)}{{\left(n+2\right)}^{2}}+\frac{n(n-4)}{2{\left(n+2\right)}^{2}},\;n>4,\\ & =\frac{3n^{2}-2n+8}{2{\left(n+2\right)}^{2}},\;n>4. \end{array}$$

According to Equation (14), n=6 and I=13/16, the robustness of CKF is poor in this situation. The non-positive covariance matrix caused by strong nonlinear or data errors may bring many problems in Kalman filters, such as filtering error or divergence, which also can lead to the error of Cholesky decomposition in CKF. In order to improve the numerical stability of CKF5, and considering that the singular value decomposition (SVD) has better numerical robustness than Cholesky [24], SVD is introduced into CKF5.

**Figure 1:**
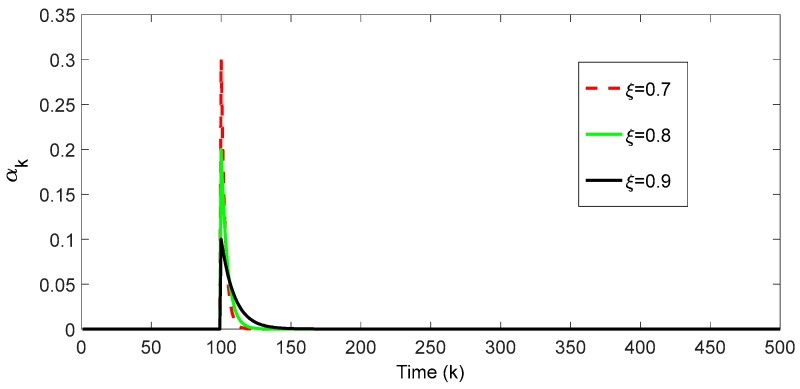
The distribution of weights of innovations under different ξ.

For s linear optimal Kalman filter, when the gain matrix $K_{k}$ is optimal, the innovation sequence ${\mathbb{Z}}_{k}$ should be orthogonal. This conclusion can be extended to nonlinear filtering. The adaptive Kalman (hereinafter referred to as AKF) scales the error covariance matrix or system noise matrix by calculating the ratio of matrix ranks of actual innovation and filtered innovation [17,18]. Essentially, the traditional AKF only partly scale the error covariance matrix in the next filtering period, and the utilization rate of innovation is low. For the innovation sequence, the contribution of the new innovation to the state estimation is large than the old one. Therefore, ICKF5 uses the exponent fading factor to weigh the innovation sequence, and then scale the entire error covariance matrix in the next filtering period. Assuming the current filter period is k and the weight set of innovation sequence is $\left\{\alpha_{i}\right\}$, we have:

(39) $$\sum_{i=1}^{k}\alpha_{i}=1,\;\alpha_{i-1}=\alpha_{i}\cdot \xi ,$$

where ξ is fading factor and ξ∈(0, 1), empirically, ξ∈(0.6, 1). To illustrate the impact of ξ on $\alpha_{i}$, ξ was taken as 0.7, 0.8, and 0.9, respectively, for the convenience of observation, the innovations are weighted by the exponent fading factor from k=100. The distribution of $\alpha_{i}$ is shown in Figure 1.

Figure 1 shows that if the ξ is big, the weight of the current innovation is small, correspondingly, the weights of old innovations are big, which means that the old innovation has bigger influence than the new innovation on calculating the covariance matrix of innovation sequence. The opposite conclusion can be obtained if ξ is small. A recursive formula for the covariance matrix of the innovation sequence ${\mathbb{Z}}_{k}$ is given below. When the forgetting factor ξ is determined, then:

(40) $$\rho_{k}=\sum_{i=1}^{k-1}\xi^{i}.$$

It follows from Equations (39) and (40) that:

(41) $$\alpha_{i}=\frac{\xi^{k-i}}{\rho_{k}},\;i=1,2,\ldots ,k.$$

The open window method is used in traditional AKF, the covariance matrix ${\hat{P}}_{\mathbb{Z},k}$ of innovation ${\mathbb{Z}}_{k}$ at time k can be expressed as:

(42) $${\hat{P}}_{\mathbb{Z},k}=\frac{1}{k-1}\sum_{i=1}^{k}{\mathbb{Z}}_{i}{\mathbb{Z}}_{i}^{T}.$$

The weight of 1/(k−1) in Equation (42) can be replaced by the weights of $\alpha_{i}$ in Equation (41), then a recursive formula between ${\hat{P}}_{\mathbb{Z},k}$ and ${\hat{P}}_{\mathbb{Z},k-1}$ can be expressed as:

(43) $$\begin{array}{cc} {\hat{P}}_{\mathbb{Z},k} & =\sum_{i=1}^{k}\frac{\xi^{k-i}}{\rho_{k}}Z_{i}Z_{i}^{T}\\ & =\frac{Z_{k}Z_{k}^{T}}{\rho_{k}}+\frac{\sum_{i=1}^{k-1}\xi^{k-i}Z_{i}Z_{i}^{T}}{\rho_{k}}\\ & =\frac{Z_{k}Z_{k}^{T}}{\rho_{k}}+\frac{\xi \rho_{k-1}}{\rho_{k}\rho_{k-1}}\sum_{i=1}^{k-1}\xi^{k-i-1}Z_{i}Z_{i}^{T}\\ & =\frac{Z_{k}Z_{k}^{T}}{\rho_{k}}+\frac{\xi \rho_{k-1}}{\rho_{k}\rho_{k-1}}\sum_{i=1}^{k-1}\xi^{k-i-1}Z_{i}Z_{i}^{T}\\ & =\frac{Z_{k}Z_{k}^{T}}{\rho_{k}}+\frac{\rho_{k}-1}{\rho_{k}}\left(\frac{1}{\rho_{k-1}}\sum_{i=1}^{k-1}\xi^{k-i-1}Z_{i}Z_{i}^{T}\right)\\ & =\frac{Z_{k}Z_{k}^{T}}{\rho_{k}}+\frac{\rho_{k}-1}{\rho_{k}}{\hat{P}}_{\mathbb{Z},k-1}. \end{array}$$

In order to improve the feedback effect of innovation, we scale the error covariance matrix of the next filtering period $\beta_{k}$ times entirely. The increase of the error covariance matrix makes the filter gain $K_{k}$ increase and the error correction ability of the innovation in the next filtering period is strengthened. This method can reduce the dependence of the filter on the model, and then improve the filtering accuracy and reduce the risk of divergence. $\beta_{k}$ is defined as:

(44) $$\beta_{k}=Max\left\{\frac{trace({\hat{P}}_{\mathbb{Z},k}-R_{k})}{trace(H_{k}P_{k|k-1}{H_{k}}^{T})},\;1\right\},$$

where trace(·) is the operator of obtaining the rank of a matrix, $R_{k}$ is the measurement error covariance matrix at time k, $H_{k}$ is the measurement matrix at time k. The error covariance matrix of next filtering period is scaled as:

(45) $$P_{k+1|k}=\beta_{k}\cdot P_{k+1|k}.$$

The algorithm of ICKF5 is summarized as follows:

Prediction phase:

(1) Input the initial values for filtering: $x_{k-1|k-1},\;P_{k-1|k-1},\;Q_{k-1},\;\beta_{k-1}=1,\;R_{k}$.
(2) Compute cubature points $x_{i},\;(i=1,\ldots ,7)$ according to Equation (26).
(3) Compute the predicted mean ${\hat{x}}_{k|k-1}$ and predicted covariance $P_{k|k-1}$ according to Equations (27) and (28), respectively.
(4) Scaling the entire $P_{k|k-1}$: $P_{k|k-1}=\beta_{k-1}\cdot P_{k|k-1}$.

Update phase:

(5) Decompose $P_{k|k-1}$ with the SVD method.
(6) Compute cubature points $x_{i}^{\prime },\;(i=1,\ldots ,7)$ according to Equation (30).
(7) Compute the estimated measurement ${\hat{z}}_{k|k-1}$, the gain matrix $K_{k}$ and the covariance matrixes of $P_{k|k-1}^{zz}$, $P_{k|k-1}^{xz}$ according to Equations (31)–(34), respectively.
(8) Update ${\hat{x}}_{k|k}$, $P_{k|k}$ and $\beta_{k}$ according to Equations (35), (36) and (44), respectively.

The scaling of the error covariance matrix by ICKF5 can effectively improve the utilization of innovation and speed up the convergence of initial alignment. ICKF5 is a kind of suboptimal algorithm, if the scaling value of $\beta_{k}$ is too large, the system will be in a state of overshoot, which may lead to oscillation or divergence. For example, when getting the initial alignment in the convergence stage, the gain $K_{k}$ and the error covariance matrix $P_{k|k-1}$ should ideally remain unchanged or little changed. If the feedback for error covariance matrix is big at the stage of alignment convergence, it may cause the filtering convergence to be unstable or lead to the divergence. In order to reduce the possibility of divergence of initial alignment filtering and ensure the stationarity after convergence, we use a short-term feedback strategy that we stop the scaling of $\beta_{k}$ in ICKF5 before the filtering occurs oscillating or diverging, which can ensure the convergence of initial alignment. For convenience, we call the short-term feedback algorithm IICKF5. The first step of IICKF5 is that the ICKF5 is used to complete the initial alignment firstly, then the gradient sequence $\nabla \psi_{i}=\mathrm{diff}(\psi_{i})$ of the set of azimuth misalignment angle $\left\{\psi_{i}\right\}$ outputted by ICKF5 can be obtained, where diff(·) is gradient operator. The second step, the time $T_{\max}$ corresponding to the maximum value of the gradient is chosen as the time to terminate the feedback to the error covariance matrix $P_{k|k-1}$. $T_{\max}$ can be expressed as:

(46) $$T_{\max}=Max\left\{abs\left\{\nabla \psi \right\}\right\}.$$

**Figure 2:**
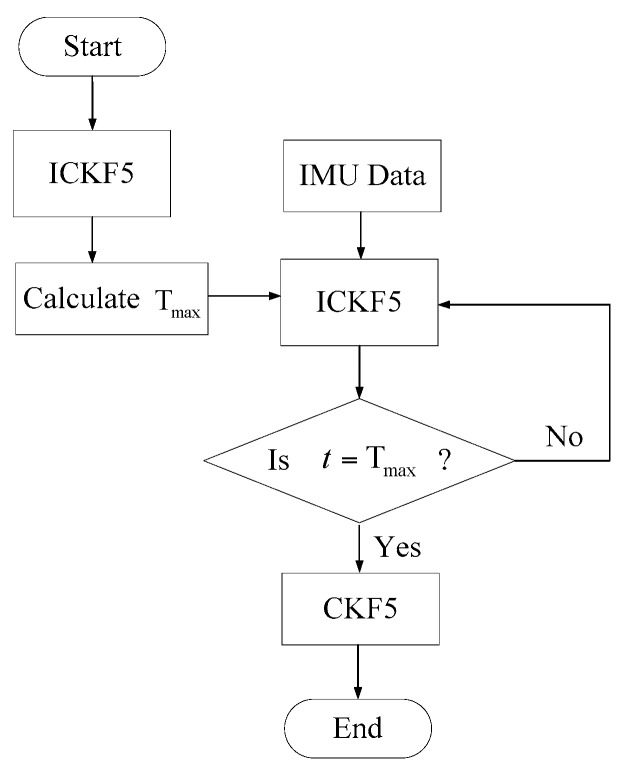
The flow chart of IICKF5.

To be brief, ICKF5 is used in the period of $\left[1,T_{\max}\right)$ and CKF5 is used in $\left[T_{\max},T_{end}\right]$, where $T_{end}$ is the end time of initial alignment filtering. The flow chart of IICKF5 is shown in Figure 2.

Compared to ICKF5, IICKF5 needs to recalculate the stored IMU data and the misalignment angle set {*x_k_*} output by ICKF5. IICKF5 has a certain delay compared with ICKF5, and some storage space is needed.

### 3.3. Simulation and Its Analysis

**Figure 3:**
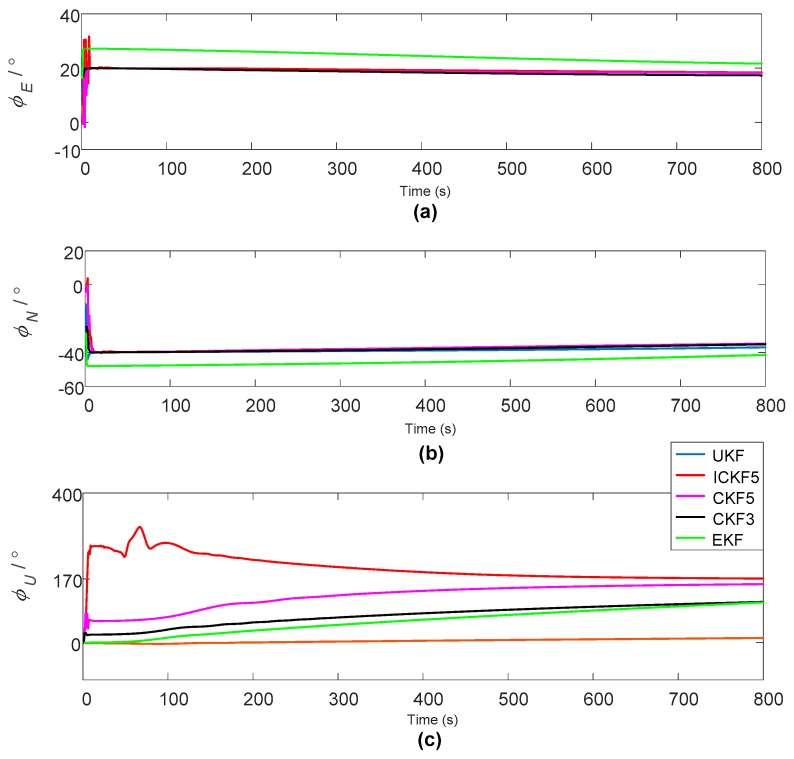
The estimated misalignment angles (ϕ(0)=[20°, −40°, 170°]): (**a**) Pitching misalignment angle; (**b**) rolling misalignment angle; (**c**) azimuth misalignment angle.

In order to verify the effectiveness of ICKF5 and IICKF5, a simulation of initial alignment under static base conditions is performed. The simulation conditions are set as follows: the gyro constant drift is $\varepsilon_{C}=0.01\;{(}^{\circ }/h)$, random drift is $\varepsilon_{N}=0.001\;{(}^{\circ }/\sqrt{h})$; the accelerometer zero bias is $\nabla_{C}=50\;\mu g$, random deviation is $\nabla_{N}=5\;(\mu g/\sqrt{h})$; the local geographic latitude is 34°, longitude is 108°; the misalignment angles are set as ϕ(0)=[20°, −40°, 170°] and the simulation period is 800 s. The general parameters of the initial alignment algorithms are set with the same values. The initial value of $X_{0}$ is set as $X_{0}=[0;0;0;0;0;0]$. The matrix of Q is determined according to the random drift of IMU, and $Q_{k}=diag{\left\{(0.001^{\circ });\;(0.001^{\circ });\;(0.001^{\circ });\;(5ug);\;(5ug);\;(5ug)\right\}}^{2}$. The initial error covariance matrix is set as $P_{0}=diag{\left([[10;10;180]\cdot \pi /180;\;[1;1;1]]\right)}^{2}$. Where diag(·) is the operator of obtaining the diagonal matrix with diagonal elements. For UKF, α=1e−3, β=2 and λ=0. EKF, UKF [25], CKF3, CKF5, ICKF5 and IICKF5 algorithms are applied to the nonlinear initial alignment. In addition to IICKF5, the calculated misalignment angles are as shown in Figure 3.

Figure 3 shows that the pitching misalignment angles converge to 20° and the rolling misalignment angles converge to −40° with the exception of EKF, and only the azimuth misalignment angles of CKF5 and ICKF5 converge to 170°.

**Figure 4:**
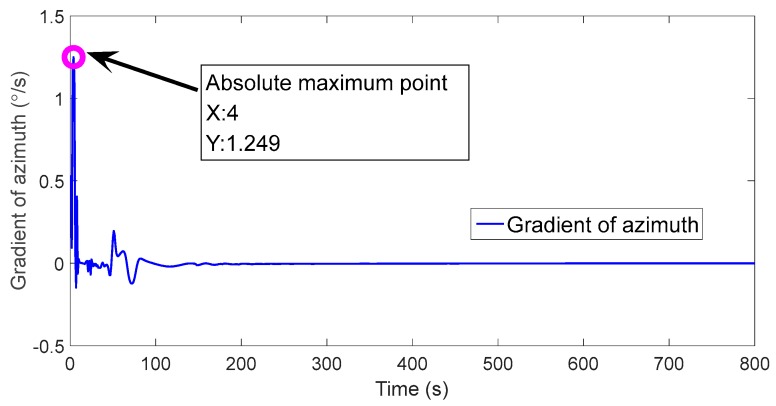
The gradient values of azimuth angle error.

The gradient values of the azimuth angle errors of ICKF5 are shown in Figure 4.

**Figure 5:**
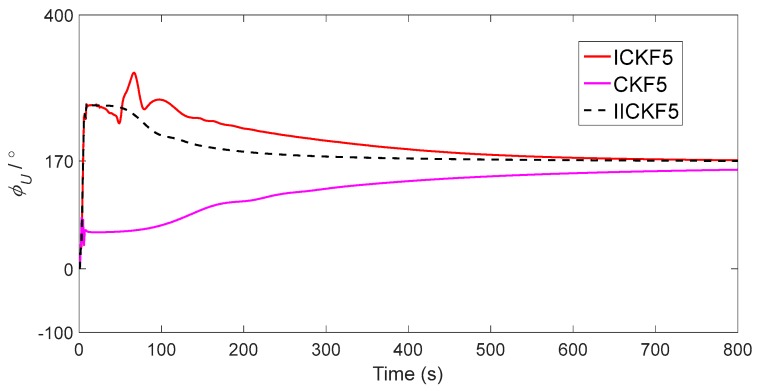
The estimated azimuth misalignment angles of CKF5, ICKF5 and IICKF5.

Figure 4 shows that there is an obvious mutation in the azimuth angle error at t=4 s, which is the overshoot caused by ICKF5, so $T_{\max}=4\;s.$ The estimated azimuth angles of CKF5, ICKF5 and IICKF5 are shown in Figure 5.

As seen in Figure 5, IICKF5 enters the convergence stage at about 400 s, while CKF5 and ICKF5 converge slower than IICKF5. Figure 3 also shows that the convergences of horizontal misalignment angles are faster than that of the azimuth misalignment angles, which is due to the fact that the observability of the horizontal misalignment angles is high under the velocity matching mode. There is a coupling relationship between the horizontal misalignment angles and the bias of accelerometer. When the variation of heading angle is small, the accuracies of the horizontal misalignment angles depend on the bias of the accelerometer. The simulation experiment in this paper is based on static base, so the variation of heading angle is very small, and the horizontal misalignment has a slow drift due to the bias of the accelerometer. From this point of view, the estimation period of the horizontal misalignment angles is not the longer the better. For the sake of accuracy, we select the estimations of horizontal misalignment angles at 50 s and select the estimation value of azimuth misalignment angle at the last moment (800 s) as the outputs of alignment, and the alignment errors are shown in Table 1.

Table 1 shows that the pitch and the azimuth misalignment errors of IICKF5 are the smallest, and the azimuth misalignment error is close to the limitd alignment precision of 0.05°, which meets the initial alignment requirement.

## 4. Dynamic Vehicle Experiment

**Figure 6:**
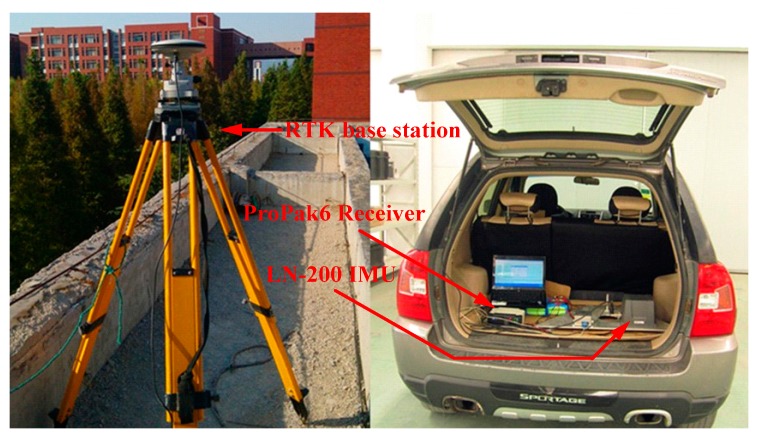
The field test setup.

The experimental equipment is shown in Figure 6. The GNSS real-time differential system consisted of two model ProPak6 receivers (NovAtel, Calgary, AB, Canada). The attitude reference system is a NovAtel SPAN system (NovAtel, Calgary, AB, Canada), which can reach centimeter level precision. An LN-200 IMU named from the Novatel Company is used for the initial alignment experiment. The bias of the accelerometer is 0.3 mg and its scale factor is 300 ppm. The constant drift, the random drift and the scale factor of gyro are $\varepsilon_{C}=1\;{(}^{\circ }/h)$, $\varepsilon_{N}=0.07{(}^{\circ }/\sqrt{h})$ and 100 ppm, respectively. The output rates of the LN-200, SPAN system and GNSS system are 200 Hz, 100 Hz and 5 Hz, respectively.

**Figure 7:**
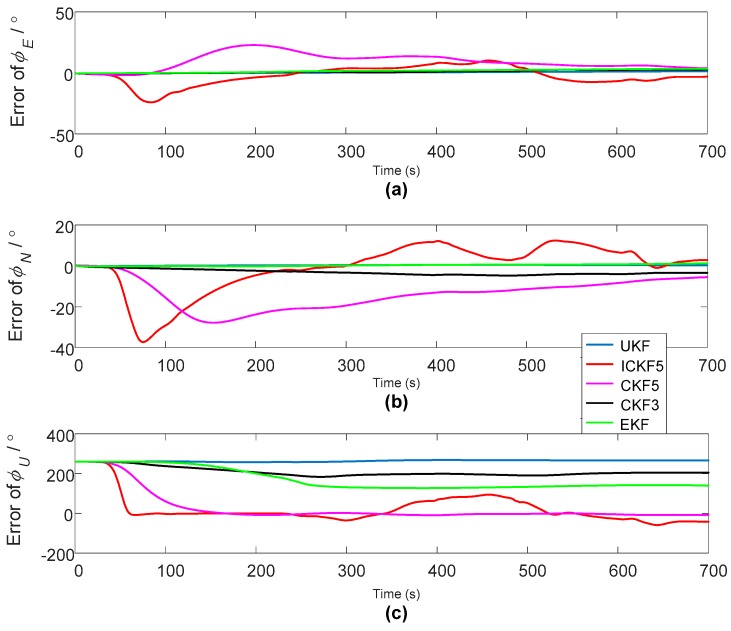
The alignment errors of large azimuth misalignment angle of dynamic vehicle experiment: (**a**) Pitch errors; (**b**) roll errors; (**c**) heading errors.

The period of initial alignment is 700 s. In addition to IICKF5, the calculated azimuth angle errors are as shown in Figure 7.

**Figure 8:**
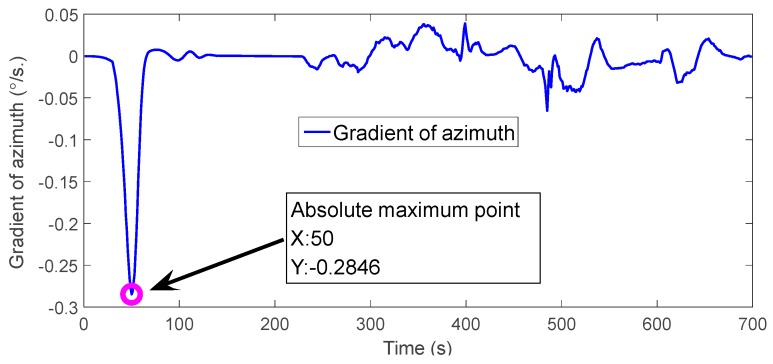
The gradient values of azimuth angle error (dynamic vehicle experiment).

For the azimuth angle errors, Figure 7 shows that EKF, UKF, CKF3 did not converge to the accurate azimuth angle, ICKF5 has faster convergence rate than CKF5, and ICKF5 has much more oscillations in the convergence process than CKF5 because of the overshoot. For the pitch angle and the roll angle errors, we can see that CKF5 and ICKF5 have greater oscillations than other algorithms, which is caused by the inaccuracy of model noises, such as the GPS’s measurement noise, IMU noise and so on. The nonlinear filter of high order precision amplifies the noises with high frequencies, which easily results in larger estimation errors if the estimated state values are small. When obtaining the gradient of the azimuth angles of ICKF5 in Figure 7c, the gradient values are as shown in Figure 8.

**Figure 9:**
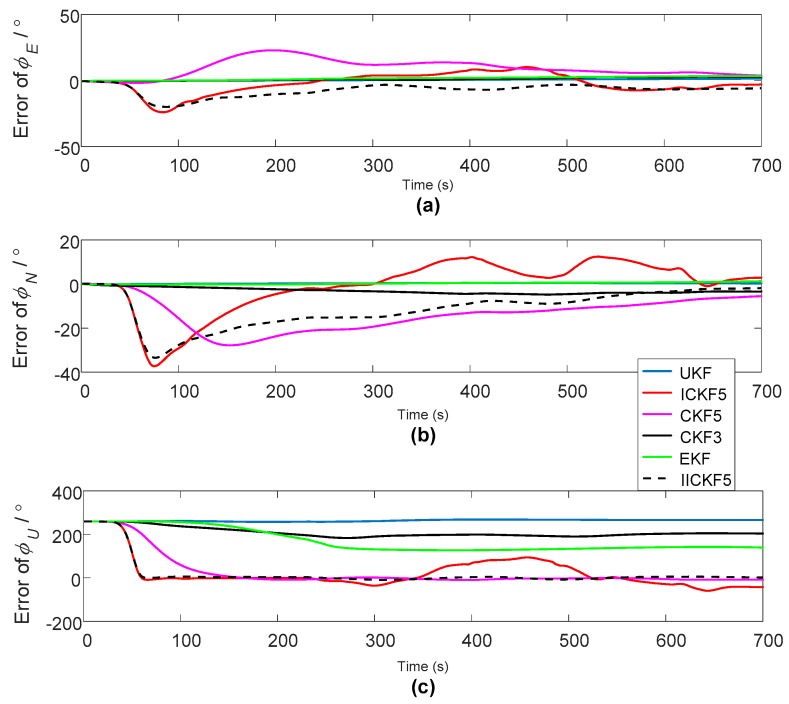
The alignment errors of different algorithms: (**a**) Pitch errors; (**b**) roll errors; (**c**) heading errors.

Figure 8 shows that there is a mutation in the azimuth angle error at t=50 s, so $T_{\max}=50\;s.$ The estimated misalignment angles of all the algorithms are shown in Figure 9.

From Figure 9 we can find that IICKF5 has a faster convergence rate than CKF5 and ICKF5 except for the pitching misalignment angle, and the azimuth misalignment angle of IICKF5 has better convergence stability than ICKF5. The alignment errors at the end of alignment (at 700 s) are shown in Table 2.

Table 2 shows that IICKF5 has better accuravy than CKF5 in estimating the rolling misalignment angle and azimuth misalignment angle. When the alignment is finished, the alignment errors of ICKF5 and IICKF5 did not converge to small values (e.g., 1°), so more time is needed for fine alignment. In order to further compare the convergence abilities of different algorithms, the mean and standard deviation of azimuth misalignment errors are calculated in the range of [100 s, 700 s], which can be regarded as a relatively complete convergence period for ICKF5 and IICKF5, and the results are shown in Table 3.

From Table 3 we can see that IICKF5 has the smallest errors of mean and standard deviation. In this sense, the improved algorithm is suitable for quickly identifying a large azimuth misalignment angle in a complex alignment environment. ICKF5 is a kind of real-time algorithm that represents an improvement on CKF5, which does not need to process historical IMU data and no storage space for the IMU is needed. IICKF5 needs to obtain the gradient value of the historical azimuth angle, so the historical IMU data need to be stored for the second initial alignment filtering. For the 700 s period of in-motion initial alignment, the length of IMU data is 70,000 (in order to synchronize with the reference system), and the storage space for IMU data is 2869 KB. The data stored are double and floating types with 64 bits. The update rate of GNSS is 5 Hz, so the storage space it needs is small, and the size is 10 KB. The capacity of the azimuth misalignment angle data is 6 KB. The total storage space needed for IICKF5 is 2885 KB, and the additional burden on the navigation computer is small.

## 5. Conclusions

It is not only convenient to calculate the innovation variance matrix in a recursive and exponent fading factor way, but also to effectively use the current and old updates. The speed and precision of convergence can be effectively improved by scaling error covariance matrix with the ratio of matrix ranks of actual updates and filter updates. In order to solve the problem of oscillation or divergence caused by excessive scaling of the error covariance matrix, the scaling scheme is terminated when the azimuth gradient reaches the maximum. The improved CKF can improve the precision of initial alignment better than the traditional CKF. The length of filtering time is also dependent on the experience, and the robustness of the improved algorithm is also worthy of further study.

## Figures and Tables

**Table 1:** Comparison of alignment errors (°).

	EKF	UKF	CKF3	CKF5	ICKF5	IICKF5
error of pitch (°)	−7.0205	0.0899	0.1270	0.1590	0.0829	0.0291
error of roll (°)	7.8234	−0.1464	−0.1634	−0.2078	−0.2949	−0.2738
error of heading (°)	62.7783	157.7361	61.2902	13.6403	−1.2458	−0.0878

**Table 2:** Comparison of alignment errors with in-motion base (°).

	EKF	UKF	CKF3	CKF5	ICKF5	IICKF5
error of pitch (°)	−3.2814	−1.2868	−2.6771	−3.7189	3.0270	5.9149
error of roll (°)	−1.2960	−0.2867	3.3751	5.4245	−2.9030	1.8357
error of heading (°)	−139.5622	−266.3212	−204.3457	7.9986	42.5004	−1.9540

**Table 3:** Mean and Standard Deviation (SD) of azimuth misalignment errors (°).

	EKF	UKF	CKF3	CKF5	ICKF5	IICKF5
Mean (°)	−155.2618	−264.1352	−201.1962	1.6920	−6.1625	−0.6852
SD (°)	38.8935	3.8062	11.5359	10.2307	41.2056	4.6229
